# Extracellular Vesicles Derived from Metastatic Melanoma Cells Transfer α7-nAChR mRNA, Thus Increasing the Surface Expression of the Receptor and Stimulating the Growth of Normal Keratinocytes

**DOI:** 10.32607/actanaturae.11734

**Published:** 2022

**Authors:** M. L. Bychkov, A. V. Kirichenko, I. N. Mikhaylova, A. S. Paramonov, M. P. Kirpichnikov, M. A. Shulepko, E. N. Lyukmanova

**Affiliations:** Shemyakin-Ovchinnikov Institute of Bioorganic Chemistry, Russian Academy of Sciences, Moscow, 117997 Russia; Moscow Institute of Physics and Technology, State University, Dolgoprudny, Moscow region, 141701 Russia; Federal State Budgetary Institution named N.N. Blokhin National Medical Research Center of Oncology of the Ministry of Healthcare of the Russian Federation, Russia, Moscow, 115548 Russia; Interdisciplinary Scientific and Educational School of Moscow University "Molecular Technologies of the Living Systems and Synthetic Biology", Faculty of Biology, Lomonosov Moscow State University, Moscow, 119234 Russia

**Keywords:** α7-nAChR, vesicles, metastatic melanoma, keratinocytes, oncotherapy, cancer

## Abstract

We have previously shown that extracellular vesicles secreted by metastatic melanoma cells stimulate the growth, migration, and stemness of normal keratinocytes. This study showed for the first time that extracellular vesicles secreted by the metastatic melanoma cell lines mel H, mel Kor, and mel P contain, both at the mRNA and protein levels, the α7-type nicotinic acetylcholine receptor (α7-nAChR), which is involved in the regulation of the oncogenic signaling pathways in epithelial cells. Incubation with the vesicles secreted by mel H cells and containing the highest amount of mRNA coding α7-nAChR increased the surface expression of α7-nAChR in normal Het-1A keratinocytes and stimulated their growth. Meanwhile, both of these effects disappeared in the presence of α-bungarotoxin, an α7-nAChR inhibitor. A bioinformatic analysis revealed a correlation between the increased expression of the CHRNA7 gene coding α7-nAChR in patients with metastatic melanoma and a poor survival prognosis. Therefore, extracellular vesicles derived from metastatic melanoma cells can transfer mRNA coding α7-nAChR, thus enhancing the surface expression of this receptor and stimulating the growth of normal keratinocytes. Targeting of α7-nAChR may become a new strategy for controlling the malignant transformation of keratinocytes.

## INTRODUCTION

Melanoma is an aggressive tumor that is formed by transformed melanocytes [1]. Melanoma progression is mediated by the secretion of extracellular vesicles (membrane-enveloped structures loaded with various proteins and nucleic acids) by tumors cells. Extracellular vesicles are involved in the transduc tion of oncogenic signals between tumor cells, as well as between the tumor and the surrounding tissues [2, 3]. Fibroblasts, immune cells, and keratinocytes regulate melanocyte physiology and control melanoma proliferation, invasion, and angiogenesis by the secretion of paracrine growth factors and intercellular communication [4, 5]. However, keratinocytes can secrete mitogenic and pro-inflammatory factors under stress conditions (e.g., under photo-induced damage) [6].

We have shown previously that extracellular vesicles secreted by metastatic melanoma cells stimulate the growth, migration, and stemness of normal keratinocytes [7]. The α7 nicotinic acetylcholine receptor (α7-nAChR) is involved in the regulation of the differentiation and growth of normal keratinocytes [8]. Its activation by nicotine or nicotine derivatives contained in tobacco (nitrosamines) promotes malignant transformation of keratinocytes [9]. However, the potential involvement of α7-nAChR in the stimulation of the keratinocyte growth induced by extracellular vesicles derived from melanoma cells has not been studied yet.

Here, we demonstrated for the first time that extracellular vesicles secreted by metastatic melanoma cells contain α7-nAChR at the mRNA and protein levels. Incubation in the presence of vesicles derived from the mel H cells increased the surface expression of α7-nAChR in normal keratinocytes and stimulated their growth; these effects were not observed in the presence of α-bungarotoxin (α-Bgtx), an α7-nAChR inhibitor. These findings provide a new insight into the role of extracellular vesicles secreted by metastatic melanoma and α7-nAChR in the malignant transformation of keratinocytes.

## EXPERIMENTAL

The metastatic melanoma cell lines mel H, mel Kor, and mel P were collected from patients at the N.N. Blokhin National Medical Research Center of Oncology, Ministry of Healthcare of the Russian Federation (Moscow, Russia), and characterized earlier [10]. The cells were grown in the RPMI-1640 medium (PanEco, Russia) supplemented with 10% fetal bovine serum (Cytiva, UK) and 1% penicillin/streptomycin (PanEco). To remove endogenous exosomes, fetal bovine serum was centrifuged (70 min, 120,000 g), filtered, and mixed with cell media. Human keratinocytes Het-1A (ATCC, USA) were cultured in the BEB medium (Lonza, Switzerland) according to the procedure described earlier [7]. Extracellular vesicles were isolated from metastatic melanoma cells using the procedure described in [7]: the cells were cultured in an exosome-depleted medium; the growth medium was centrifuged sequentially at 10,000 g (15 min, 4°C) and 120,000 g (70 min, 4°C). Protein complexes were removed by gel filtration using the Superdex G-250 resin (GE Healthcare, USA). Vesicle size was estimated by the dynamic light scattering (DLS) method using the DynaPro Titan instrument (Wyatt Technology, USA). Expression of the exosomal marker TSG101 in the vesicles was confirmed by Western blotting.

The nAChR subunit mRNA expression was analyzed by real-time PCR according to the procedure described earlier in [7]. Expression of the CHRNA3, CHRNA4, CHRNA5, CHRNA7, CHRNA9, CHRNB2, and CHRNB4 genes (primers are listed in Table) was analyzed using a Roche LightCycler 96 amplificator (Roche, Switzerland). The mRNA level was normalized to the expression of S18 ribosomal RNA.

The presence of α7-nAChR in the extracellular vesicles at the protein level was analyzed by Western blotting [7]. After the gel electrophoresis and transfer of vesicle lysates, nitrocellulose membranes were blocked with 5% milk and incubated with primary anti-TSG101 (1 : 1000, ABIN2780037, Antibodies-Online, Germany) or anti-α7-nAChR rabbit antibodies (1 : 1000, ABIN5611363, Antibodies- Online) at 4°C overnight, washed, and incubated with HRP-conjugated anti-rabbit antibodies (1 : 5000, 111-035-003, Jackson Immunoresearch, USA) for 1 h at 20°C. The membranes were then washed, and the HRP signal was registered using the ECL substrate (Bio-Rad, USA) and an ImageQuant LAS 500 camera system (GE Healthcare, USA).

To study the effect of extracellular vesicles on keratinocyte proliferation, the cells were seeded in 96-well plates (5 × 10^3^ cells/well); after 24 h, they were supplemented with vesicles (total protein concentration 50 μg/ml) and/or 10 μM α-bungarotoxin (α-Bgtx, an α7-nAChR inhibitor, Tocris, UK) and additionally incubated for 72 h without media replacement. The concentration of the total vesicular protein corresponded to that in the plasma of the cancer patients (20–100 μg/mL) [7]. Cell viability was analyzed using a WST-1 colorimetric assay (Santa Cruz, USA) [11]. The data were normalized to averaged data in the control wells containing untreated cells.

**Table:** The primers used in this study

Gene	Primer		Amplicon size, bp
	Forward	Reverse	
S18 SSU RNA	CTC AAC ACG GGA AAC CTC AC	CGC TCC ACC AAC TAA GAA CG	110
CHRNA3	TGT CCC TCT CTG CTT TGT CAC	CCC AGG TTC TTG ATC GGA TGT T	169
**CHRNA4**	**TCG TCC TCT ACA ACA AGT GAG**	**GGT CCA GGA GCC GAA TTT CA**	**199**
CHRNA5	CGT CTG GTT GAA ACA GGA ATG G	ACA GTG CCA TTG TAC CTG ATG A	185
CHRNA7	TTT ACA GTG GAA TGT GTC AGA	TGT GGA ATG TGG CGT CAA G	88
CHRNA9	GGA GGC CAG ACA TCG TCT TA	CAC TGC TGG TTG TCA AAA GGG	168
CHRNB2	ATC TCC TGG ATC CTT CCC GC	AGA AGG ACA CCT CGT ACA TGC C	290
CHRNB4	CGC CTT CCC TGG TCC TTT TC	TGT TCA CAC CCT CGT AGC GG	381

The effect of the vesicles and α-Bgtx on α7-nAChR expression in keratinocytes was studied after staining the cells with TRITC-labeled α-Bgtx (Sigma- Aldrich, USA) using an Attune NxT flow cytometer (Life Technologies, USA) and the procedure described earlier [11]. The median fluorescence was normalized to the autofluorescence of unstained cells.

The correlation between the CHRNA7 expression level in patients with metastatic melanoma from the TCGA database (the SKCM study) and the prognosis of their survival was analyzed using the Xena software [12].

## RESULTS AND DISCUSSION

Extracellular vesicles secreted by melanoma cells contain microRNA, mRNA, and proteins that stimulate the proliferation, migration, and stemness of normal keratinocytes [7]. However, the recruitment of nAChRs, which regulate many oncogenic processes in epithelial cells, into these effects of extracellular vesicles has not been studied previously.

**Fig. 1:**
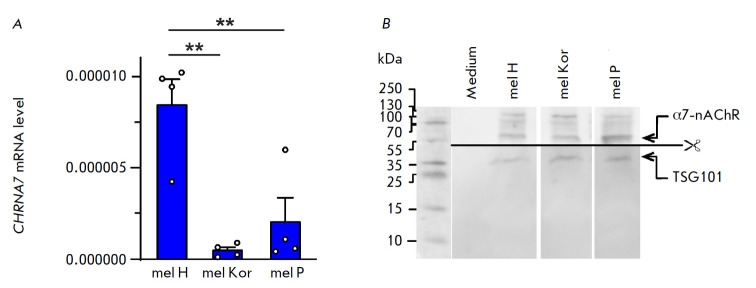
Analysis of the α7-nAChR expression in extracellular vesicles derived from metastatic melanoma cells. (*A*) – Analysis of the *CHRNA7 *expression in extracellular vesicles derived from mel H, mel Kor, and mel P cells. Expression of mRNA was assayed by real-time PCR and normalized to the S18 ribosomal RNA. The data are presented as the mean mRNA level ± SEM (n = 4). ** (*p * < 0.01) indicates a significant difference between the data groups according to one-way ANOVA, followed by the Tukey’s post hoc test. (*B*) – Analysis of α7-nAChR protein expression in extracellular vesicles derived from mel H, mel Kor, and mel P cells by Western blotting. TSG101 was used as an exosomal marker

We have demonstrated by real-time PCR for the first time that extracellular vesicles secreted by patient-derived metastatic melanoma cells mel H, mel Kor, and mel P contain CHRNA7 mRNA encoding the α7-nAChR subunit of the homopentameric receptor (Fig. 1A). The highest CHRNA7 expression level was observed for vesicles secreted by mel H cells. Meanwhile, no mRNA encoding the α3, α4, α5, α9, β2, and β4 subunits of nAChR were detected. Western blotting proved that the vesicles derived from all the studied melanoma cell lines contained the α7-nAChR protein (Fig. 1B). Interestingly, the previous analysis of the protein composition of extracellular vesicles secreted by primary melanomas had detected no α7-nAChR [3]. Expression of this receptor can possibly be a specific feature of extracellular vesicles derived from metastatic melanoma.

**Fig. 2:**
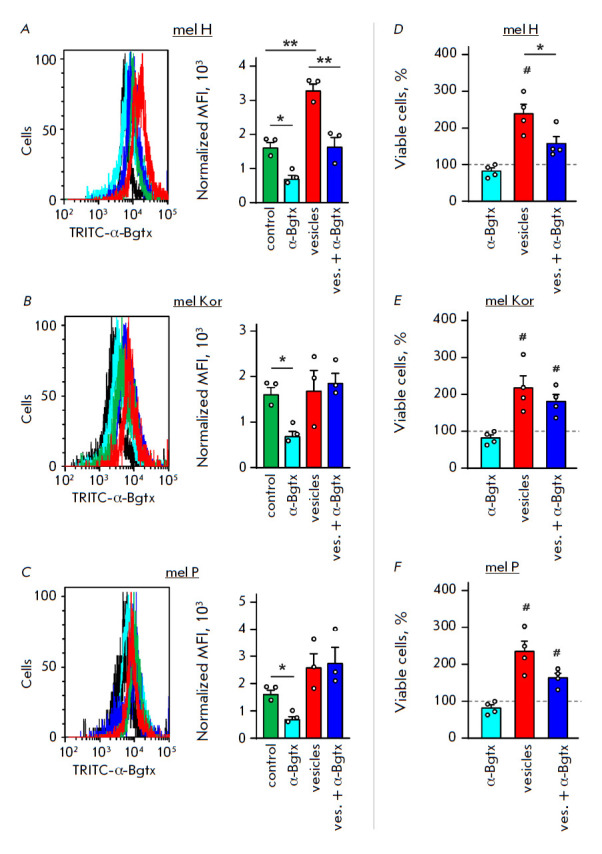
Analysis of the effects of extracellular vesicles secreted by metastatic melanoma cells and α-Bgtx on the α7-nAChR expression and keratinocyte proliferation. *A–C *– Expression of α7-nAChR on the surface of normal keratinocytes incubated in the presence of extracellular vesicles derived from mel H (*A*), mel Kor (*B*), and mel P (*C*) cells and/or α-Bgtx. The data are presented as normalized median fluorescence (MFI) ± SEM (n = 3). * (*p * < 0.05) and ** (*p * < 0.01) indicate a significant difference between the data groups according to one-way ANOVA, followed by the Tukey’s post hoc test. *D–F *– The effects of extracellular vesicles derived from mel H (*D*), mel Kor (*E*), and mel P (*F*) cells and/or α-Bgtx on the proliferation of normal keratinocytes. The data are % of untreated cells ± SEM (n = 4). # (*p * < 0.05) indicates a significant difference from the untreated cells according to the one-sample t-test. * (*p * < 0.05) indicates a significant difference between the data groups according to one-way ANOVA, followed by the Tukey post hoc test

We have shown earlier that extracellular vesicles secreted by metastatic melanoma mel P contain mRNA encoding the epidermal growth factor receptor (EGFR), and that incubation of normal keratinocytes in the presence of these vesicles leads to upregulated EGFR expression on the keratinocyte surface and stimulates their proliferation [7]. Here, we studied the effect of extracellular vesicles derived from metastatic melanoma mel H, mel Kor, and mel P cells on the α7-nAChR expression in normal keratinocytes. Flow cytometry revealed that only incubation in the presence of extracellular vesicles derived from mel H cells causes a statistically significant upregulation of the α7-nAChR expression on the surface of normal keratinocytes. Treatment of keratinocytes with vesicles derived from mel Kor and mel P had no effect on the expression level of the receptor (Fig. 2A, B, C). The results are consistent with the PCR data showing that the highest CHRNA7 expression level is actually observed in vesicles derived from mel H cells (Fig. 1A). It is plausible that vesicles derived from metastatic melanoma cells mel H transfer mRNA encoding α7-nAChR to keratinocytes, thus increasing the expression of this receptor in normal cells. Interestingly, incubation in the presence of α-Bgtx, an inhibitor of α7-nAChR, reduced the expression of this receptor on the keratinocyte surface (Fig. 2A–C) both in the presence and absence of vesicles derived from mel H cells, pointing to some positive feedback between the receptor activity and its expression.

In all the cases, incubation with vesicles derived from the mel H, mel Kor, and mel P cells significantly increased the number of viable keratinocytes (Fig. 2D–F). However, α-Bgtx cancelled the mitogenic effect induced only by mel H-derived vesicles and this correlates with the fact that incubation of keratinocytes with vesicles from mel Kor and mel P caused no changes in the α7-nAChR expression in keratinocytes (Fig. 2B,C). It is noteworthy that incubation with α-Bgtx in the absence of vesicles did not significantly reduce the number of viable keratinocytes (Fig. 2D–F), although the toxin significantly reduced the expression of the receptor (Fig. 2A–C). This indicates that keratinocyte growth is independent of the α7-nAChR regulation under normal conditions, but transfer of the CHRNA7 gene by the vesicles from mel H cells significantly increases the receptor expression in keratinocytes, thus additionally stimulating their proliferation. It seems that, although expression of the α7 receptor is comparable in all the analyzed types of vesicles (Fig. 1B), CHRNA7 mRNA is the principal transferred component that stimulates keratinocyte growth in the presence of vesicles. Other factors unrelated to α7-nAChR (e.g., EGFR mRNA) are probably responsible for the increased keratinocyte proliferation observed upon incubation with vesicles from the mel Kor and mel P cells [7].

**Fig. 3:**
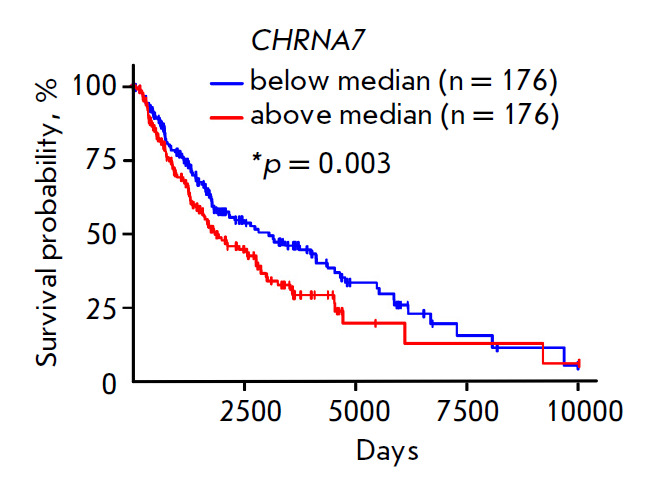
Bioinformatic analysis of the correlation between the survival of patients with metastatic melanoma and the *CHRNA7 *expression level. Patients were divided into two groups with the gene expression levels above and below the median value. Statistical analysis of patient survival was performed by the Kaplan–Meier method using the log-rank test

In order to understand how the CHRNA7 expression level can affect the development of oncogenic processes and, particularly, correlate with cell malignancy, we performed a bioinformatic analysis of the expression of this receptor in biopsy specimens taken from patients with metastatic melanoma. A Kaplan– Meier analysis showed that the upregulated CHRNA7 expression icorrelates with an unfavorable survival prognosis in patients with metastatic melanoma (Fig. 3). Our findings indicate that α7-nAChR is potentially involved in the pathogenesis of metastatic melanoma, and that transfer of mRNA encoding this receptor within extracellular components can be a mechanism responsible for the stimulation of tumor progression.

## CONCLUSIONS

Expression of α7-nAChR, both at the mRNA and protein levels, was detected for the first time in extracellular vesicles secreted by different lines of metastatic melanoma cells. Extracellular vesicles derived from the mel H cells demonstrating the highest CHRNA7 expression were shown to transfer receptor mRNA to normal keratinocytes, thus increasing the α7-nAChR expression on their surface and stimulating their growth. Since no such effect of vesicles derived from the mel H cells was observed in the presence of α-Bgtx, it is a promising strategy to target α7-nAChR to control the malignant transformation of normal keratinocytes.

## Abbreviations

α7-nAChR: α7 nicotinic acetylcholine receptor
BEBM: bronchial epithelial cell growth basal medium
α-Bgtx: α-bungarotoxin
HRP: horseradish peroxidase
WST-1: water-soluble tetrazolium salt 1
